# Multiple sclerosis and breast cancer risk: a meta-analysis of observational and Mendelian randomization studies

**DOI:** 10.3389/fninf.2023.1154916

**Published:** 2023-05-03

**Authors:** Tian Fang, Zhihao Zhang, Huijie Zhou, Wanchun Wu, Liqun Zou

**Affiliations:** ^1^Department of Medical Oncology, Cancer Center, West China Hospital, Sichuan University, Chengdu, China; ^2^Department of Thyroid Breast Surgery, Xi’an No.3 Hospital, The Affiliated Hospital of Northwest University, Xi’an, Shaanxi, China

**Keywords:** multiple sclerosis, breast cancer, Mendelian randomization, meta-analysis, genetic variants

## Abstract

**Background:**

Several observational studies have explored the relationships between multiple sclerosis (MS) and breast cancer; however, whether an association exists remains unknown.

**Methods:**

We conducted a meta-analysis of observational studies and Mendelian randomization (MR) based on genetic variants to identify the relationship between MS and breast cancer. The observational studies were searched from PubMed, Embase, Web of Science, and Scopus to assess the relationship between MS and breast cancer from inception to 07 Nov 2022. Moreover, we explored the association between genetically pre-disposed MS and breast cancer risk based on an MR study. The summary analysis for MS from two separate databases [International Multiple Sclerosis Genetics Consortium (IMSGC), FinnGen] and the summary analysis for breast cancer from Breast Cancer Association Consortium.

**Results:**

Fifteen cohort studies involving 173,565 female MS patients were included in this meta-analysis. The correlation between MS and breast cancer was not statistically significant [relative ratio (RR) = 1.08, 95% confidence interval (CI) = 0.99–1.17]. In the MR analysis, we did not observe causal associations of genetically determined MS with breast cancer and its subtypes from both the IMSGC and FinnGen datasets.

**Conclusion:**

The meta-analysis of observational and MR based on genetic variants does not support the correlation between MS and breast cancer.

## 1. Introduction

Multiple sclerosis (MS) is a chronic autoimmune demyelinating disease (Anderson et al., 2021), and several experimental studies have found an imbalance between the inflammatory and regulatory T-cell balance in MS patients (Stephens et al., 2009; Quinn and Axtell, 2018). Because the immune system plays an initial role in MS and cancer, it is reasonable to suspect that patients with MS may have an increased incidence of cancer.

Breast cancer is the most frequent malignant tumor worldwide and is a serious threat to women’s lives and health (Sung et al., 2021). If we can identify high-risk factors for breast cancer and enhance screening, we can detect breast cancer early and thus improve the survival rate. Many researchers have explored the relationship between MS and breast cancer. Groome et al. (2022) reported a study that compared the incidence of breast cancer and colorectal cancer in MS patients through a population-based study, and Groome et al. (2022) concluded that MS patients are easily detected to have colorectal cancer but not breast cancer. Bosco-Lévy et al. (2022) found that the risk of breast cancer was higher among MS patients than among population controls (HR = 1.12, 95% CI = 1.03−1.23). Those consistencies may be caused by different study methodologies, designs, or unadjusted confounding factors (Bosco-Lévy et al., 2022). Hence, we performed a Mendelian randomization (MR) study, a new etiological investigation method, combined with previous observational studies to explore the epidemiological relationship between MS and breast cancer.

MR can identify the causal effect between risk factors and health outcomes using genetic variants as instrumental variables (Bowden and Holmes, 2019). MR can provide robust results between exposures and outcomes because mitosis allows for the random allocation of gametes to offspring, which allows for results that are not confounded by potential confounding factors and allows for a better search for the cause of the disease compared to traditional observational studies (Richmond and Davey Smith, 2022). Our study aimed to explore whether MS is a risk or protective factor for breast cancer by performing an observational meta-analysis and MR study.

## 2. Materials and methods

### 2.1. Meta-analysis

#### 2.1.1. Search strategy

**FIGURE 1:**
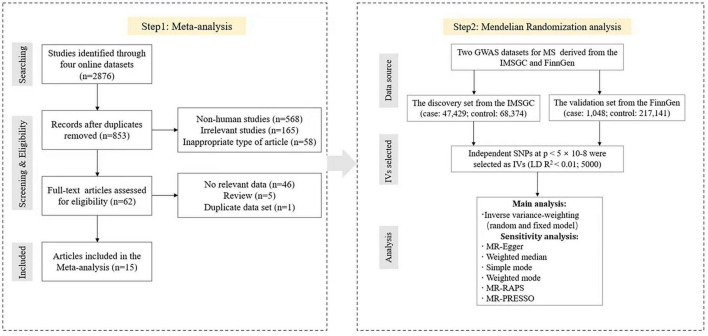
Flow chart for meta-analysis and Mendelian randomization analysis.

The overall design of this study is shown in Figure 1. We searched four online databases, PubMed, Embase, Web of Science, and Scopus, for articles published in English up to 23 September 2022. The search keywords were (“multiple sclerosis”) and (“breast neoplasms” or “breast cancer” or “breast tumor”).

#### 2.1.2. Inclusion and exclusion criteria

Studies that met the following criteria were included in our meta-analysis: (i) cohort studies of patients with MS and (ii) all published articles were in English. (iii) evaluated the association or risk between MS and breast cancer. (iv) sample size not less than 300. (v) Studies provided odds ratios (ORs), relative risks (RRs), hazard ratios (HRs), or standardized incidence ratios (SIRs) with their 95% confidence intervals (CIs) of breast cancer among MS patients. The exclusion criteria were as follows: (i) systematic review, case report, letter, meeting or meta-analysis; and (ii) insufficient data to obtain effect sizes.

#### 2.1.3. Data extraction and assessment of quality

Two researchers (Zhang and Fang) extracted the data independently. When disagreements arose, they were resolved through discussion. We extracted the following information: the name of the first author, country, year of publication, sample size, study period (including follow-up), and effect size (OR, RR, HR or SIR) with 95% CI. Two researchers (Fang and Zhang) evaluated the quality of the included studies using the nine-item Newcastle−Ottawa Quality scale. We have set a minimum follow-up period of 5 years, and no points will be awarded for less than 5 years or for not reporting the follow-up period. Concerning completeness of the follow-up, we set 5% as a cutoff level of loss during the follow-up. Those with a quality score of no more than 5 will be excluded.

#### 2.1.4. Data synthesis

We extracted the effect size, if available, or calculated it from available data, including OR, RR, HR, or SIR. SIRs are calculated as the ratio of the number of observed cases to the number of expected cases in the exposed population. The 95% CI for log(SIR) was constructed *via* the term + 1.96/[square root (O)], where O was the observed case (Alder et al., 2006). Since the absolute risk of breast cancer is low, the four types of measures are expected to have similar estimates of RR. Consequently, our final summary results are presented in the form of RR (Larsson et al., 2007). The same statistical methods have been applied in other meta-analysis articles (Siristatidis et al., 2013).

We used STATA version 14.0 (Stata Corporation, College Station, TX, USA) software for data analysis and visualization. Statistical significance is expressed as a pooled *P*, and a *P* < 0.05 was considered statistically significant. The Cochran *Q*-test and I2 statistics were used to measure heterogeneity; *P* < 0.1 and I^2^ value > 50% represented substantial heterogeneity (Higgins et al., 2003). The random model was used if strong heterogeneity existed, and subgroup analysis was conducted to detect potential heterogeneity. If there is no heterogeneity, the fixed-effects model will be used (DerSimonian and Laird, 2015). Sensitivity analysis was used to check data stability, and Egger’s test was used to detect publication bias.

### 2.2. Mendelian randomization

#### 2.2.1. Study design

We explored the relationship between MS and breast cancer using a two-sample MR study, which applied genetic predictors of exposure to outcome (Emdin et al., 2017). Our MR study had to follow the following three assumptions: (1) There is a strong association between the instrument variants (IVs) and the risk factor. (2) There are no associations between the IVs and any confounders. (3) IVs should not influence outcomes by confounders or other ways (Davey Smith and Hemani, 2014).

#### 2.2.2. Instrument selection

Genome-wide association studies (GWAS) of MS were derived from the International Multiple Sclerosis Genetics Consortium (IMSGC) and FinnGen datasets. In brief, the IMSGC analyzed genetic data from 15 GWASs, including 47,429 MS cases and 68,374 controls, all of whom were of European ancestry (International Multiple Sclerosis Genetics Consortium, 2019). The summary data of IMSGC were adjusted for confounding factors, age, sex, immunomodulatory drugs, batch effects, and the first 10 principal components (International Multiple Sclerosis Genetics Consortium, 2019). The diagnosis of MS was defined by the International Classification of Diseases (ICD) from the Finnish R5 release dataset, and there were 1,048 cases and 217,141 controls of Finnish ancestry. Sex, age, and 10 principal components were logistic regression covariates, and the data can also be found in MRCIEU datasets (finn-b-G6_MS). The independent single nucleotide polymorphisms (SNPs) at *p* < 5 × 10^–8^ were selected as IVs (linkage disequilibrium: *R*2 < 0.01; 5,000). We assessed the correlation of each IV with risk factors using the F statistic, with *F* < 10 representing a weak instrumental variable and exclusion (Pierce et al., 2011).

#### 2.2.3. Data source of outcome

The summary GWAS of breast cancer was derived from the Breast Cancer Association Consortium (BCAC) with 122,977 cases (ER + breast cancer, 69,501; ER- breast cancer, 21,468) and 105,974 controls of European ancestry (Michailidou et al., 2017). Two genotyping arrays were used for genotyping: iCOGS arrays in 40,178 breast cancer cases and 35,314 controls^1^ and OncoArray in 68,242 cases and 52,367 controls^2^. OncoArray and iCOGS were adjusted for country and study, respectively.

Ethical approval and consent for the summary statistics were obtained from the original publication.

#### 2.2.4. Statistical analysis

The Wald ratio was used to estimate the effect of exposure on the outcome for each SNP. All effects of SNPs were meta-analyzed using the inverse-variance weighted (IVW) method. The IVW method was used as the main analysis to assess the association between MS and breast cancer in our MR study, and both fixed-effect and random-effect models were performed. Moreover, MR−Egger regression, weighted median, weighted mode, simple mode, and robust adjusted profile score (MR-RAPS) were applied to detect the robustness of our results (Bowden et al., 2017).

Several analyses were performed to check heterogeneity and pleiotropy. Cochrane’s *Q*-value was used to assess heterogeneity. The MR−Egger method was based on the Instrument Strength Independent of Direct Effect (InSIDE) assumption, and the value of the intercept term is far from zero, indicating horizontal pleiotropy (Bowden et al., 2015). The weighted median is more accurate when more than half of the IVs are valid (e.g., due to pleiotropy) (Bowden et al., 2016). MR pleiotropy residual sum and outlier (MR-PRESSO) was used to detect any outlier SNPs and potential horizontal pleiotropy (Verbanck et al., 2018).

### 2.3. Statistical analysis

All statistical analyses were performed using R software (version 4.1.1) and STATA 12.0. A *P*-value of <0.05 was considered statistically significant. The “TwoSampleMR,” “mr.raps,” and “MRPRESSO” packages were applied in our MR study. F analysis was used to detect weak instrument variants (*F* < 10). Was used R^2^ to estimate the ability of instruments variants present the exposure.

## 3. Results

### 3.1. Meta-analyses

#### 3.1.1. Characteristics and quality assessment of the included studies

The literature search with PubMed, Embase, Web of Science, and Scopus yielded 2,876 studies. After removal of duplicates, 853 studies remained, and 62 full-text articles were reviewed after screening. Finally, 15 cohort studies were enrolled in our study, which included 173,565 female MS patients (Midgard et al., 1996; Sumelahti et al., 2004; Achiron et al., 2005; Nielsen et al., 2006; Bahmanyar et al., 2009; Hemminki et al., 2012; Kingwell et al., 2012; Sun et al., 2014; Hajiebrahimi et al., 2016; Etemadifar et al., 2017; Nørgaard et al., 2019; Grytten et al., 2020; Johnson et al., 2021; Marrie et al., 2021; Bosco-Lévy et al., 2022; Mariottini et al., 2022). The quality scores of all studies were no more than 6. The flow diagram for the included and excluded studies is shown in Figure 1, and the reasons for exclusion are listed accordingly. The characteristics of all included studies are shown in Table 1.

**TABLE 1:** Characteristics of the included studies.

References	Type of measure	Country	Number of MS (female)	Cancer in MS	Cancer in control	Study period (including follow up)	Main measurements	NOS
Achiron et al., 2005	SIR	Israel	892	15/892 = 1.68%	Expected 15.4/892 = 1.73%	1960–2003	SIR (0.97; 0.59–1.62)	6
Bahmanyar et al., 2009	HR	Sweden	13,218	451/13,218 = 3.41%	5,174/132,638 = 3.90%	1969–2005	HR (0.95; 0.86–1.05)	7
Etemadifar et al., 2017	SIR	Iran	1,330	11/1,330 = 0.83%	Expected 6.2/1,330 = 0.47%	2006–2014	SIR (1.77; 1.12–2.76)	6
Grytten et al., 2020	HR	Norway	4,597	160/4,597 = 3.48%	836/25,268 = 3.31%	1952–2016	HR (1.11;0.94–1.32)	8
Hemminki et al., 2012	SIR	Swedish	8,486	223/8,486 = 2.62%	Expected NR	1964–2008	SIR (1.05; 0.92–1.20)	7
Kingwell et al., 2012	SIR	UK	4,998	110/4,998 = 2.20%	Expected NR	1980–2004	SIR (0.94; 0.77–1.33)	8
Bosco-Lévy et al., 2022	HR	France	69,142	1,027/69,142 = 1.49%	916/69,142 = 1.32%	2008–2015	HR (1.12;1.03–1.23)	6
Mariottini et al., 2022	RR	Italy	452	6/452 = 1.33%	71/4,520 = 1.57%	2002–2018	RR (0.85;0.37–1.94)	7
Marrie et al., 2021	HR	Canada	37,767	NR	NR	1994–2017	HR (0.92;0.78–1.09)	7
Midgard et al., 1996	SIR	Norway	741	21/741 = 2.83%	Expected 12.33/741 = 1.66%	1953–1992	SIR (1.70; 1.05–2.60)	6
Hajiebrahimi et al., 2016	HR	Sweden	19,330	474/19,330 = 2.45%	4,756/193,461 = 2.46%	1968–2012	HR (1.08; 0.98–1.19)	7
Nielsen et al., 2006	RR	Danish	3,318	51/3,318 = 1.54%	14,682/1,510,467 = 0.97%	1968–1997	RR (1.54;1.17–2.03)	6
Nørgaard et al., 2019	SIR	Denmark	7,258	111/7,258 = 1.53%	Expected 113/7,258 = 1.56%	1995–2016	SIR (0.98;0.81–1.18)	6
Sumelahti et al., 2004	SIR	Finland	1,050	17/1,095 = 1.55%	Expected 21.3/1,095 = 1.95%	1967–1999	SIR (0.80;0.50–1.13)	6
Sun et al., 2014	HR	Taiwan	986	12/986 = 1.22%	27/3,944 = 0.68%	1997–2010	HR (2.23; 1.11–4.46)	8

MS, multiple sclerosis; OR, studies provided odds ratios; RR, relative risks, HR: hazard ratios; SIR, standardized incidence ratios; NR, not reported; NOS, Newcastle–Ottawa scale.

#### 3.1.2. Risk of breast cancer among MS patients

**FIGURE 2:**
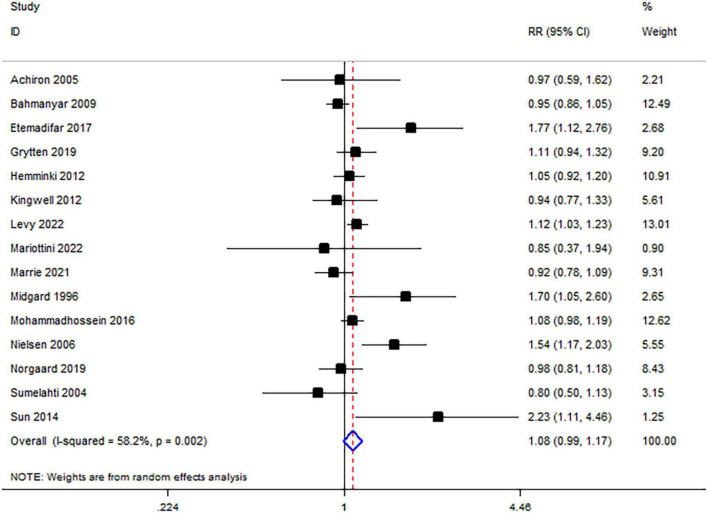
Meta-analysis results of the association between MS and breast cancer.

The forest plot shows the results of this meta-analysis (Figure 2), and the results indicated that there was no relationship between MS and breast cancer [relative ratio (RR) = 1.08, 95% confidence interval (CI): 0.99–1.17]. A random-effects model was performed because of significant heterogeneity among those studies (*I*^2^ = 58.2%, *p* = 0.002). Several subgroup analyses were conducted to detect the potential sources of significant heterogeneity.

**FIGURE 3:**
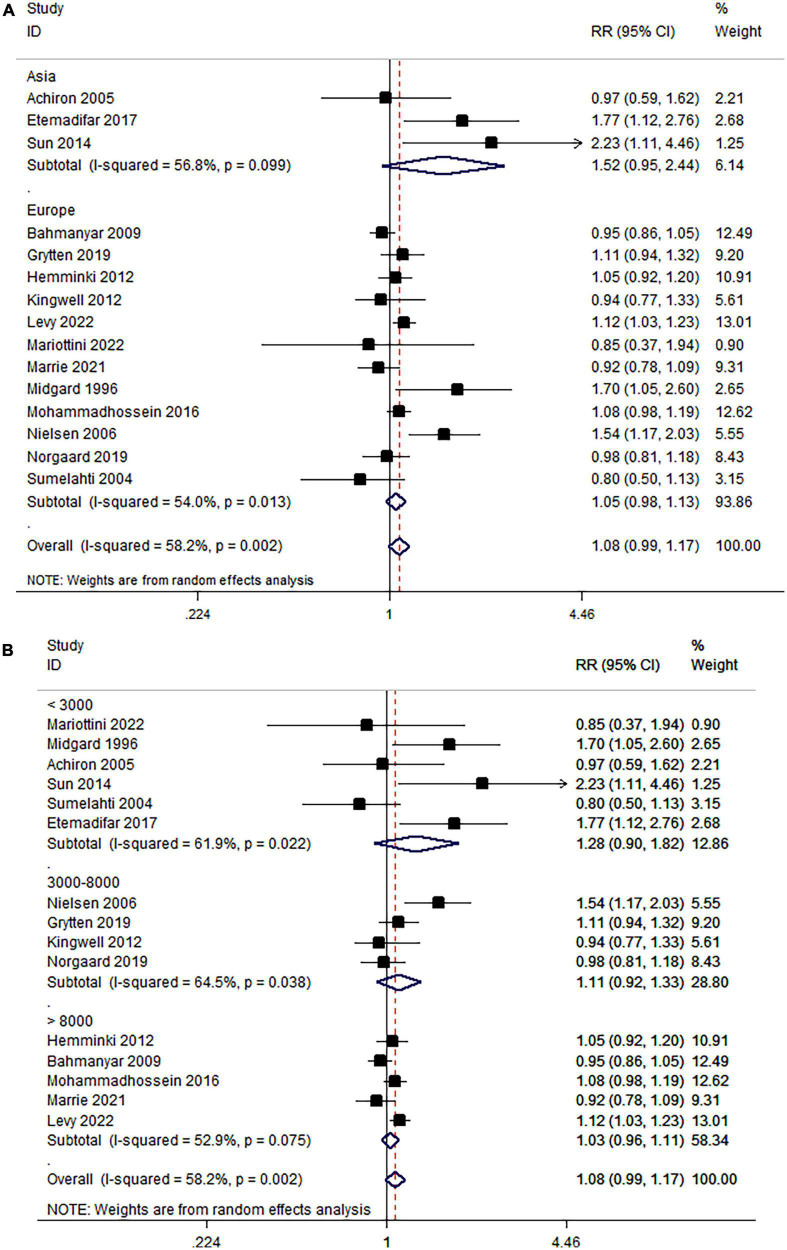
**(A)** Association of MS and breast cancer in subgroups stratified by ancestries and **(B)** number of MS females.

Subgroup analysis concerning ancestries did not find an association between MS and breast cancer in Asia or Europe (Asia: RR = 1.52, 95% CI: 0.95–2.44; Europe: RR = 1.05, 95% CI = 0.98–1.13) (Figure 3A). The subgroup analysis based on the number of MS females revealed no relationship between MS and breast cancer (<3,000: RR = 1.28, 95% CI: 0.90–1.82; 3,000–7,000: RR = 1.11, 95% CI = 0.92–1.33; >7,000: RR = 1.03, 95% CI = 0.96–1.33) (Figure 3B). Subgroup analysis did not detect the source of heterogeneity.

#### 3.1.3. Sensitivity analysis

The results did not show any evidence of publication bias (Begg’s Test 0.553; Egger’s Test = 0.297) (Supplementary Figures 1, 2). Leave-one-out sensitivity analysis indicated that the results were robust (Supplementary Figure 3).

### 3.2. MR analysis

#### 3.2.1. The results based on the IMSGC dataset

The detailed SNP information is shown in Supplementary Table 1. The F analysis for all SNPs was more than 10, which indicated that no weak instrument variants were detected. The *R*^2 *values*^ were 27.3, 27.4, and 26.7% for overall breast cancer, ER+ breast cancer and ER− breast cancer, respectively. There was no evidence that MS is related to overall breast cancer (IVW-fixed OR: 1.001, 95% CI: 0.990–1.024; IVW-random OR: 1.001, 95% CI: 0.991–1.023), ER+ breast cancer (IVW-fixed OR: 1.008, 95% CI: 0.990–1.028; IVW-random OR: 1.008, 95% CI: 0.989–1.029) and ER− breast cancer (IVW-fixed OR: 0.995, 95% CI: 0.968–1.023; IVW-random OR: 0.995, 95% CI: 0.975–1.016) based on the discovery set from the IMSGC dataset, with similar results estimated in other sensitivity analyses (Table 2). MR−Egger and Cochrane’s *Q*-value results indicated that there was no pleiotropy in this part, and the results are also shown in Table 3. No outliers were detected by performing the MR-PRESSO test for all the estimates.

**TABLE 2:** MR estimates for the causal effect of multiple sclerosis on breast cancer.

Outcome	Method	IMSGC				FinnGen			
		**SNPs**	**OR**	**95% CI**	***P***	**SNPs**	**OR**	**95% CI**	***P***
Overall BC	IVW-fixed	53	1.001	0.990–1.024	0.384	10	1.000	0.990–1.010	0.970
	IVW-random	53	1.001	0.991–1.023	0.410	10	1.000	0.987–1.013	0.976
	MR-Egger	53	1.012	0.934–1.097	0.768	10	1.006	0.981–1.033	0.641
	WM	53	1.012	0.989–1.036	0.294	10	1.004	0.990–1.017	0.606
	Simple mode	53	1.051	0.992–1.113	0.097	10	0.992	0.969–1.015	0.511
	Weighted mode	53	1.021	0.967–1.077	0.463	10	1.006	0.991–1.021	0.474
	MR-RAPS	53	1.008	0.989–1.026	0.413	10	0.999	0.997–1.001	0.919
ER + BC	IVW-fixed	52	1.008	0.990–1.028	0.371	10	1.006	0.994–1.019	0.322
	IVW-random	52	1.008	0.989–1.029	0.398	10	1.006	0.990–1.023	0.463
	MR-Egger	52	1.060	0.963–1.167	0.239	10	1.017	0.984–1.050	0.347
	WM	52	1.007	0.980–1.036	0.608	10	1.007	0.990–1.022	0.474
	Simple mode	52	0.989	0.919–1.065	0.769	10	0.981	0.939–1.026	0.428
	Weighted mode	52	0.994	0.932–1.062	0.868	10	1.018	1.000–1.036	0.079
	MR-RAPS	52	1.007	0.986–1.029	0.503	10	1.003	0.982–1.025	0.788
ER − BC	IVW-fixed	55	0.995	0.968–1.023	0.735	10	0.987	0.968–1.006	0.180
	IVW-random	55	0.995	0.975–1.016	0.645	10	0.987	0.971–1.004	0.128
	MR-Egger	55	1.019	0.881–1.179	0.803	10	0.978	0.942–1.014	0.260
	WM	55	0.986	0.949–1.025	0.471	10	0.979	0.955–1.004	0.099
	Simple mode	55	0.973	0.892–1.063	0.551	10	0.992	0.954–1.031	0.689
	Weighted mode	55	0.982	0.904–1.067	0.668	10	0.983	0.957–1.009	0.228
	MR-RAPS	55	0.993	0.964–1.023	0.632	10	0.988	0.969–1.008	0.238

MR, Mendelian randomization; IVW, inverse variance weighting; WM, weighted median; MR-RAPS, robust adjusted profile score; BC, breast cancer; ER + BC, estrogen positive breast cancer; ER − BC, estrogen negative breast cancer; AM, any migraine; OR, odds ratio.

**TABLE 3:** Heterogeneity and horizontal pleiotropy analyses results.

Outcome	IMSGC				FinnGen			
	**P_(Heterogeneity)_**	**Egger intercept**	**P_(Pleiotropy)_**	**P_(Global test)_**	**P_(Heterogeneity)_**	**Egger intercept**	**P_(Pleiotropy)_**	**P_(Global test)_**
Overall BC	0.266	−0.0007	0.896	0.266	0.096	−0.0041	0.574	0.135
ER + BC	0.257	−0.0064	0.304	0.226	0.060	−0.0064	0.485	0.105
ER − BC	0.997	−0.0029	0.651	0.998	0.639	0.0059	0.563	0.665

BC, breast cancer; ER + BC, estrogen-positive breast cancer; ER − BC, estrogen-negative breast cancer. P_(Heterogeneity)_: *p*-value of Cochrane’s *Q*-value in heterogeneity test; P_(Pleiotropy)_: the *P*-value for the intercept in the MR−Egger regression was used to present pleiotropy (*p* < 0.05): P_(Global test)_: the *P*-value for the global test in MR-PRESSO.

#### 3.2.2. The results based on the FinnGen dataset

Ten SNPs were selected as instrument variants (Supplementary Table 2). The F ranged from 29.6 to 259.2. In addition, MS was not a risk or protective factor for overall breast cancer (IVW-fixed OR: 1.000, 95% CI: 0.990–1.010; IVW-random OR: 1.000, 95% CI: 0.987–1.013), ER+ breast cancer (IVW-fixed OR: 1.006, 95% CI: 0.994–1.019; IVW-random OR: 1.006, 95% CI: 0.990–1.023) or ER- breast cancer (IVW-fixed OR: 0.987, 95% CI: 0.968–1.006; IVW-random OR: 0.987, 95% CI: 0.971–1.004) based on the validation set from FinnGen, with similar results assessed in other sensitivity analyses (Table 2). No heterogeneity or pleiotropy results were estimated in this MR study, and the results are also shown in Table 3. No outliers were detected by performing the MR-PRESSO test for all the estimates.

## 4. Discussion

The etiology of MS is unclear, but it is currently thought to be an immune-mediated demyelinating disease involving the central nervous system (Mariottini et al., 2022; Stampanoni Bassi et al., 2022). MS and breast cancer share some common features; most notably, the incidence of both diseases is much higher in women than in men (O’Malley et al., 2015). Numerous studies have explored the relationship between them, but whether MS affects the incidence of breast cancer remains controversial (Hajiebrahimi et al., 2016; Zecca et al., 2021; Groome et al., 2022). We tried to find the association between MS and breast cancer risk through meta-analysis, and finally, we included fifteen cohort studies. However, these studies were significantly different in terms of ancestry, treatment, and other factors. To avoid these confounding factors, we used Mendelian randomization as a supplement. Ultimately, Mendelian instrumental variables were obtained in 47,429 MS patients and validated in another population including 1,048 MS patients.

Fifteen cohort studies from thirteen countries were included in our meta-analysis, and five of them reported a significant relationship between MS and breast cancer risk. Our meta-analysis found that there was no relationship between MS and breast cancer risk (meta-RR = 1.08; 95% CI = 0.99–1.17; *P* = 0.073). Consistently, a meta-analysis of the relationship between MS and breast cancer conducted by Catalá-López and Tobías (2010), including only five cohort studies (meta-SIR = 1.02; 95% CI = 0.75–1.40). Our updated meta-analysis included 10 additional studies and therefore had increased statistical power. Moreover, Lopez’s meta-analysis study exhibited strong heterogeneity (*I*^2^ = 75.3%), and a moderate degree of heterogeneity was found in our study (*I*^2^ = 58.2%). The meta-regression did not find the source of heterogeneity. We attempted multiple subgroup analyses but also failed to find a source of heterogeneity, suggesting that the results might be affected by other potential confounders, such as environmental exposure and treatment.

To overcome the inherent limitations of traditional observational articles, such as difficult-to-detect confounders, reverse causation, and various biases (Davey Smith and Hemani, 2014; Burgess et al., 2017), we conducted a two-sample MR method by genetic variants to further investigate the association between MS and the risk of breast cancer. MR analysis is a new epidemiological method that uses genetic variation as a tool to explore the relationship between risk factors and outcomes (Davies et al., 2018). Genetic variation as an instrument variation must meet three basic conditions: (1) Genetic variations are closely associated with exposure. (2) Genetic variations are not associated with confounding factors. (3) Genetic variations do not directly influence outcomes (Davey Smith and Hemani, 2014). The first hypothesis is easier to prove, an F statistic greater than 10 proves that there is no instrumental variable, and all SNPs in our study have an F statistic greater than 10. The proof of the second and third hypotheses is to examine horizontal pleiotropy, which is the focus and difficulty of MR research (Hemani et al., 2018). Several different sensitivity analyses were used to detect and correct for any potential pleiotropic effects on outcomes in our study. The *P*-value of the intercept of MR−Egger proves that there is no horizontal pleiotropy, and all sensitivity analysis and IVW method results demonstrated that MS was not associated with breast cancer risk. The same conclusion was obtained in replication practice from FinnGen consortia. The lack of detected heterogeneity suggests that the results of our MR study are relatively stable.

A hypothesis to explain the risk of breast cancer identified that chronic inflammation caused by MS may result in weakened activation of the immune system or immune protection against cancer becoming protumorigenic (Hofer et al., 2010). However, some people hold a different view: MS breaks myelin by upregulating immune system activity, which may have a protective effect against cancer. Immune-inflammation-associated helper T1 cells produce large amounts of antitumor factors that help prevent cancer proliferation (Fletcher et al., 2010). Therefore, speculation that it affects breast cancer risk by affecting the immune system remains controversial. A genotyping study found that mutations in the BRCA1 gene are very close to the MS gene, which may be why there are more cases of breast cancer in MS patients than in non-MS cohorts (Holzmann et al., 2013). Of course, this study was a single-family study, so the results may not be very significant. Another problem with this acceptance is that mutations in the BRCA1 gene account for only a small fraction of the causes of breast cancer (Paul and Paul, 2014). Furthermore, drug treatment, not the disease itself, may be the real cause of cancer (Bahmanyar et al., 2009; Dolladille et al., 2021; Bosco-Lévy et al., 2022). The development of disease-modifying therapies (MDTs) has revolutionized the treatment of MS (Florou et al., 2020). However, the modulation of innate immune mechanisms and suppression of the immune system induced by the use of DMT contribute to an increased risk of malignancies, such as breast cancer, lymphoma, and melanoma, in patients treated with these drugs for long periods of time (Conzett et al., 2011; Ragonese et al., 2017; Melamed and Lee, 2019). In addition, the protective role of CD20 B cells and cytotoxic T cells plays an important role in preventing the development of malignancies, and anti-CD20 therapy for MS has been found to play an important role in the development of cancer (Kelsey et al., 2021). A study found that cancer rates are three times higher among MS patients who receive immunotherapy than among those who never receive immunotherapy. This study found a particular trend toward breast cancer and cancer in the digestive tract, urinary tract, and skin (Lebrun et al., 2008). However, the 15 studies we included did not indicate whether patients with MS received the MDT or other types of treatment, which prevented us from performing subgroup analyses by treatment modality, which is a limitation of our study.

Several advantages of our study exist. First, our meta-analysis included fifteen cohort studies with a large study population (173,565 female patients with MS), providing more reliable results than previous meta-analyses (13,419 female patients with MS) (Catalá-López and Tobías, 2010). Second, this is the first MR study to estimate the causal relationship between MS and breast cancer. Our study design strictly followed the three assumptions of MR (VanderWeele et al., 2014). Third, we used two completely different cohorts of MS in our MR study, and the primary results and sensitivity analysis showed a robust conclusion, all of which suggest that the outcome was stable.

In conclusion, the results of this large MR study and meta-analysis do not support an association of MS with breast cancer risk, and our conclusion may provide insights for future studies.

## Data availability statement

The original contributions presented in this study are included in the article/Supplementary material, further inquiries can be directed to the corresponding author.

## Author contributions

TF and ZZ designed this study and performed the data analyses. TF conducted the analyses and drafted the manuscript. LZ directed the analytical strategy and supervised the study from conception to completion. TF, HZ, and WW revised the manuscript draft. All authors contributed to the interpretation of data and critically revised the manuscript.

## Footnotes

1
https://ccge.medschl.cam.ac.uk/research/consortia/icogs/

2
https://bcac.ccge.medschl.cam.ac.uk/bcacdata/oncoarray/oncoarray-and-combined-summary-result/

## Supplementary material

Supplementary Figure 1 Funnel plot of Begg’s test for publication bias.

Supplementary Figure 2 Funnel plot of Egger’s test for publication bias.

Supplementary Figure 3 Sensitivity analysis of the leave-one-out test for the meta-analysis.
