# Supplementary material for: Multiple sclerosis and breast cancer risk: a meta-analysis of observational and Mendelian randomization studies
Source: Front Neuroinform. 2023 May 3;17:1154916. doi: 10.3389/fninf.2023.1154916 (PMC10191210; doi:10.3389/fninf.2023.1154916)
Supplement: Supplementary file 1 [file Table_1.DOCX]

**Supplementary Table 1.** Information on instrumental variables of MS from IMSGC database.

| Outcome | SNP | CHR | EA | OA | EAF | BETA | SE | P | F | R^2^(%) |
| --- | --- | --- | --- | --- | --- | --- | --- | --- | --- | --- |
| Overall BC | rs11083862 | 19 | A | T | 0.81 | 0.101 | 0.017 | 4.10E-09 | 35.3 | 0.31% |
| Overall BC | rs11079784 | 17 | C | T | 0.32 | 0.107 | 0.016 | 4.30E-11 | 44.7 | 0.50% |
| Overall BC | rs2248137 | 20 | G | C | 0.26 | -0.112 | 0.017 | 7.80E-11 | 43.4 | 0.48% |
| Overall BC | rs72928038 | 6 | G | A | 0.02 | -0.161 | 0.025 | 9.00E-11 | 41.5 | 0.10% |
| Overall BC | rs1177228 | 2 | G | A | 0.92 | 0.107 | 0.019 | 8.60E-09 | 31.7 | 0.17% |
| Overall BC | rs34695601 | 14 | T | C | 0.99 | 0.109 | 0.02 | 3.20E-08 | 29.7 | 0.02% |
| Overall BC | rs6670198 | 1 | C | T | 0.22 | -0.145 | 0.018 | 2.00E-16 | 64.9 | 0.72% |
| Overall BC | rs12622670 | 2 | T | C | 0.38 | 0.107 | 0.017 | 1.00E-10 | 39.6 | 0.54% |
| Overall BC | rs1323292 | 1 | A | G | 0.98 | 0.124 | 0.022 | 1.40E-08 | 31.8 | 0.06% |
| Overall BC | rs55858457 | 7 | G | T | 0.45 | -0.113 | 0.02 | 1.20E-08 | 31.9 | 0.63% |
| Overall BC | rs35703946 | 16 | G | A | 0.89 | 0.173 | 0.029 | 1.90E-09 | 35.6 | 0.59% |
| Overall BC | rs4808760 | 19 | G | C | 0.81 | -0.135 | 0.019 | 4.80E-13 | 50.5 | 0.56% |
| Overall BC | rs1465697 | 19 | C | T | 0.31 | -0.124 | 0.019 | 3.50E-11 | 42.6 | 0.66% |
| Overall BC | rs12365699 | 11 | G | A | 0.98 | 0.144 | 0.023 | 3.10E-10 | 39.2 | 0.08% |
| Overall BC | rs4896153 | 6 | A | T | 0.33 | -0.138 | 0.019 | 1.60E-13 | 52.8 | 0.84% |
| Overall BC | rs1250551 | 10 | G | T | 0.12 | -0.116 | 0.017 | 2.70E-11 | 46.6 | 0.28% |
| Overall BC | rs28834106 | 19 | T | C | 0.29 | 0.129 | 0.02 | 3.80E-11 | 41.6 | 0.69% |
| Overall BC | rs2150879 | 17 | G | A | 0.9 | 0.104 | 0.016 | 3.30E-10 | 42.3 | 0.19% |
| Overall BC | rs28703878 | 8 | A | G | 0.57 | -0.134 | 0.021 | 4.50E-10 | 40.7 | 0.88% |
| Overall BC | rs140522 | 22 | C | T | 0.53 | -0.111 | 0.018 | 2.80E-10 | 38.0 | 0.61% |
| Overall BC | rs9992763 | 4 | T | G | 0.84 | -0.09 | 0.016 | 4.50E-08 | 31.6 | 0.22% |
| Overall BC | rs10936602 | 3 | T | C | 0.76 | 0.108 | 0.019 | 1.70E-08 | 32.3 | 0.43% |
| Overall BC | rs10801908 | 1 | C | T | 0.23 | 0.215 | 0.026 | 3.50E-16 | 68.4 | 1.64% |
| Overall BC | rs6032662 | 20 | C | T | 0.02 | 0.134 | 0.018 | 2.80E-13 | 55.4 | 0.07% |
| Overall BC | rs6589706 | 11 | A | G | 0.07 | 0.104 | 0.017 | 2.20E-09 | 37.4 | 0.14% |
| Overall BC | rs12925972 | 16 | C | T | 0.48 | 0.095 | 0.017 | 3.10E-08 | 31.2 | 0.45% |
| Overall BC | rs354033 | 7 | G | A | 0.78 | 0.108 | 0.019 | 1.20E-08 | 32.3 | 0.40% |
| Overall BC | rs405343 | 16 | G | T | 0.38 | -0.118 | 0.022 | 4.70E-08 | 28.8 | 0.66% |
| Overall BC | rs1112718 | 10 | A | G | 0.55 | 0.106 | 0.017 | 2.50E-10 | 38.9 | 0.56% |
| Overall BC | rs6990534 | 8 | A | G | 0.7 | -0.107 | 0.018 | 3.60E-09 | 35.3 | 0.48% |
| Overall BC | rs9610458 | 22 | T | C | 0.35 | 0.114 | 0.017 | 4.60E-12 | 45.0 | 0.59% |
| Overall BC | rs12478539 | 2 | G | C | 0.8 | 0.123 | 0.019 | 4.40E-11 | 41.9 | 0.48% |
| Overall BC | rs802730 | 6 | T | C | 0.9 | 0.114 | 0.018 | 3.20E-10 | 40.1 | 0.23% |
| Overall BC | rs1077667 | 19 | C | T | 0.88 | 0.152 | 0.021 | 8.40E-13 | 52.4 | 0.49% |
| Overall BC | rs1014486 | 3 | C | T | 0.3 | 0.105 | 0.016 | 1.40E-10 | 43.1 | 0.46% |
| Overall BC | rs35486093 | 1 | A | G | 0.15 | -0.179 | 0.028 | 1.60E-10 | 40.9 | 0.82% |
| Overall BC | rs4325907 | 3 | T | C | 0.46 | -0.099 | 0.017 | 3.70E-09 | 33.9 | 0.49% |
| Overall BC | rs17724508 | 16 | T | C | 0.96 | 0.213 | 0.038 | 2.60E-08 | 31.4 | 0.35% |
| Overall BC | rs6496663 | 15 | A | C | 0.67 | -0.101 | 0.018 | 2.80E-08 | 31.5 | 0.45% |
| Overall BC | rs62420820 | 6 | A | G | 0.1 | 0.137 | 0.019 | 2.50E-13 | 52.0 | 0.34% |
| Overall BC | rs3809627 | 16 | A | C | 0.65 | -0.097 | 0.018 | 3.20E-08 | 29.0 | 0.43% |
| Overall BC | rs11256593 | 10 | T | C | 0.13 | 0.186 | 0.017 | 6.80E-27 | 119.7 | 0.78% |
| Overall BC | rs9591325 | 13 | T | C | 0.96 | 0.212 | 0.034 | 4.20E-10 | 38.9 | 0.35% |
| Overall BC | rs7222450 | 17 | A | G | 0.43 | 0.098 | 0.017 | 1.80E-08 | 33.2 | 0.47% |
| Overall BC | rs2317231 | 1 | G | T | 0.38 | 0.101 | 0.017 | 1.90E-09 | 35.3 | 0.48% |
| Overall BC | rs13327021 | 3 | C | T | 0.28 | -0.113 | 0.017 | 4.50E-11 | 44.2 | 0.51% |
| Overall BC | rs438613 | 3 | C | T | 0.11 | 0.138 | 0.017 | 9.40E-17 | 65.9 | 0.37% |
| Overall BC | rs35540610 | 2 | C | T | 0.26 | 0.135 | 0.019 | 2.90E-12 | 50.5 | 0.70% |
| Overall BC | rs11749040 | 5 | G | A | 0.23 | -0.197 | 0.023 | 3.50E-17 | 73.4 | 1.37% |
| Overall BC | rs57116599 | 2 | A | G | 0.82 | -0.12 | 0.02 | 2.60E-09 | 36.0 | 0.43% |
| Overall BC | rs12133753 | 1 | C | T | 0.83 | 0.128 | 0.022 | 8.50E-09 | 33.9 | 0.46% |
| Overall BC | rs1026916 | 17 | G | A | 0.64 | -0.13 | 0.017 | 1.00E-13 | 58.5 | 0.78% |
| Overall BC | rs12434551 | 14 | A | T | 0.66 | 0.104 | 0.016 | 1.80E-10 | 42.3 | 0.49% |
| Overall BC | rs9955954 | 18 | G | A | 0.74 | -0.11 | 0.019 | 1.50E-08 | 33.5 | 0.47% |
| Overall BC | rs9843355 | 3 | G | A | 0.81 | 0.134 | 0.021 | 4.70E-10 | 40.7 | 0.55% |
| ER+ BC | rs11079784 | 17 | C | T | 0.32 | 0.107 | 0.016 | 4.30E-11 | 44.7 | 0.50% |
| ER+ BC | rs7855251 | 9 | T | C | 0.38 | 0.11 | 0.02 | 4.20E-08 | 30.3 | 0.57% |
| ER+ BC | rs59655222 | 1 | T | C | 0.85 | 0.123 | 0.019 | 3.80E-11 | 41.9 | 0.39% |
| ER+ BC | rs1465697 | 19 | C | T | 0.31 | -0.124 | 0.019 | 3.50E-11 | 42.6 | 0.66% |
| ER+ BC | rs34695601 | 14 | T | C | 0.99 | 0.109 | 0.02 | 3.20E-08 | 29.7 | 0.02% |
| ER+ BC | rs354033 | 7 | G | A | 0.78 | 0.108 | 0.019 | 1.20E-08 | 32.3 | 0.40% |
| ER+ BC | rs1077667 | 19 | C | T | 0.88 | 0.152 | 0.021 | 8.40E-13 | 52.4 | 0.49% |
| ER+ BC | rs12365699 | 11 | G | A | 0.98 | 0.144 | 0.023 | 3.10E-10 | 39.2 | 0.08% |
| ER+ BC | rs11256593 | 10 | T | C | 0.13 | 0.186 | 0.017 | 6.80E-27 | 119.7 | 0.78% |
| ER+ BC | rs11083862 | 19 | A | T | 0.81 | 0.101 | 0.017 | 4.10E-09 | 35.3 | 0.31% |
| ER+ BC | rs2327586 | 6 | T | C | 0.61 | 0.118 | 0.019 | 7.80E-10 | 38.6 | 0.66% |
| ER+ BC | rs9992763 | 4 | T | G | 0.84 | -0.09 | 0.016 | 4.50E-08 | 31.6 | 0.22% |
| ER+ BC | rs28703878 | 8 | A | G | 0.57 | -0.134 | 0.021 | 4.50E-10 | 40.7 | 0.88% |
| ER+ BC | rs12478539 | 2 | G | C | 0.8 | 0.123 | 0.019 | 4.40E-11 | 41.9 | 0.48% |
| ER+ BC | rs2150879 | 17 | G | A | 0.9 | 0.104 | 0.016 | 3.30E-10 | 42.3 | 0.19% |
| ER+ BC | rs140522 | 22 | C | T | 0.53 | -0.111 | 0.018 | 2.80E-10 | 38.0 | 0.61% |
| ER+ BC | rs6990534 | 8 | A | G | 0.7 | -0.107 | 0.018 | 3.60E-09 | 35.3 | 0.48% |
| ER+ BC | rs1177228 | 2 | G | A | 0.92 | 0.107 | 0.019 | 8.60E-09 | 31.7 | 0.17% |
| ER+ BC | rs35703946 | 16 | G | A | 0.89 | 0.173 | 0.029 | 1.90E-09 | 35.6 | 0.59% |
| ER+ BC | rs12622670 | 2 | T | C | 0.38 | 0.107 | 0.017 | 1.00E-10 | 39.6 | 0.54% |
| ER+ BC | rs2317231 | 1 | G | T | 0.38 | 0.101 | 0.017 | 1.90E-09 | 35.3 | 0.48% |
| ER+ BC | rs6670198 | 1 | C | T | 0.22 | -0.145 | 0.018 | 2.00E-16 | 64.9 | 0.72% |
| ER+ BC | rs1112718 | 10 | A | G | 0.55 | 0.106 | 0.017 | 2.50E-10 | 38.9 | 0.56% |
| ER+ BC | rs405343 | 16 | G | T | 0.38 | -0.118 | 0.022 | 4.70E-08 | 28.8 | 0.66% |
| ER+ BC | rs57116599 | 2 | A | G | 0.82 | -0.12 | 0.02 | 2.60E-09 | 36.0 | 0.43% |
| ER+ BC | rs72928038 | 6 | G | A | 0.02 | -0.161 | 0.025 | 9.00E-11 | 41.5 | 0.10% |
| ER+ BC | rs55858457 | 7 | G | T | 0.45 | -0.113 | 0.02 | 1.20E-08 | 31.9 | 0.63% |
| ER+ BC | rs28834106 | 19 | T | C | 0.29 | 0.129 | 0.02 | 3.80E-11 | 41.6 | 0.69% |
| ER+ BC | rs9610458 | 22 | T | C | 0.35 | 0.114 | 0.017 | 4.60E-12 | 45.0 | 0.59% |
| ER+ BC | rs1250551 | 10 | G | T | 0.12 | -0.116 | 0.017 | 2.70E-11 | 46.6 | 0.28% |
| ER+ BC | rs10801908 | 1 | C | T | 0.23 | 0.215 | 0.026 | 3.50E-16 | 68.4 | 1.64% |
| ER+ BC | rs35486093 | 1 | A | G | 0.15 | -0.179 | 0.028 | 1.60E-10 | 40.9 | 0.82% |
| ER+ BC | rs9843355 | 3 | G | A | 0.81 | 0.134 | 0.021 | 4.70E-10 | 40.7 | 0.55% |
| ER+ BC | rs4325907 | 3 | T | C | 0.46 | -0.099 | 0.017 | 3.70E-09 | 33.9 | 0.49% |
| ER+ BC | rs1014486 | 3 | C | T | 0.3 | 0.105 | 0.016 | 1.40E-10 | 43.1 | 0.46% |
| ER+ BC | rs1323292 | 1 | A | G | 0.98 | 0.124 | 0.022 | 1.40E-08 | 31.8 | 0.06% |
| ER+ BC | rs4808760 | 19 | G | C | 0.81 | -0.135 | 0.019 | 4.80E-13 | 50.5 | 0.56% |
| ER+ BC | rs438613 | 3 | C | T | 0.11 | 0.138 | 0.017 | 9.40E-17 | 65.9 | 0.37% |
| ER+ BC | rs12133753 | 1 | C | T | 0.83 | 0.128 | 0.022 | 8.50E-09 | 33.9 | 0.46% |
| ER+ BC | rs35540610 | 2 | C | T | 0.26 | 0.135 | 0.019 | 2.90E-12 | 50.5 | 0.70% |
| ER+ BC | rs802730 | 6 | T | C | 0.9 | 0.114 | 0.018 | 3.20E-10 | 40.1 | 0.23% |
| ER+ BC | rs10936602 | 3 | T | C | 0.76 | 0.108 | 0.019 | 1.70E-08 | 32.3 | 0.43% |
| ER+ BC | rs12434551 | 14 | A | T | 0.66 | 0.104 | 0.016 | 1.80E-10 | 42.3 | 0.49% |
| ER+ BC | rs13327021 | 3 | C | T | 0.28 | -0.113 | 0.017 | 4.50E-11 | 44.2 | 0.51% |
| ER+ BC | rs17724508 | 16 | T | C | 0.96 | 0.213 | 0.038 | 2.60E-08 | 31.4 | 0.35% |
| ER+ BC | rs3809627 | 16 | A | C | 0.65 | -0.097 | 0.018 | 3.20E-08 | 29.0 | 0.43% |
| ER+ BC | rs1026916 | 17 | G | A | 0.64 | -0.13 | 0.017 | 1.00E-13 | 58.5 | 0.78% |
| ER+ BC | rs9955954 | 18 | G | A | 0.74 | -0.11 | 0.019 | 1.50E-08 | 33.5 | 0.47% |
| ER+ BC | rs7222450 | 17 | A | G | 0.43 | 0.098 | 0.017 | 1.80E-08 | 33.2 | 0.47% |
| ER+ BC | rs6496663 | 15 | A | C | 0.67 | -0.101 | 0.018 | 2.80E-08 | 31.5 | 0.45% |
| ER+ BC | rs11749040 | 5 | G | A | 0.23 | -0.197 | 0.023 | 3.50E-17 | 73.4 | 1.37% |
| ER+ BC | rs62420820 | 6 | A | G | 0.1 | 0.137 | 0.019 | 2.50E-13 | 52.0 | 0.34% |
| ER+ BC | rs12925972 | 16 | C | T | 0.48 | 0.095 | 0.017 | 3.10E-08 | 31.2 | 0.45% |
| ER+ BC | rs9591325 | 13 | T | C | 0.96 | 0.212 | 0.034 | 4.20E-10 | 38.9 | 0.35% |
| ER- BC | rs3809627 | 16 | A | C | 0.65 | -0.097 | 0.018 | 3.20E-08 | 29.0 | 0.43% |
| ER- BC | rs2327586 | 6 | T | C | 0.61 | 0.118 | 0.019 | 7.80E-10 | 38.6 | 0.66% |
| ER- BC | rs1014486 | 3 | C | T | 0.3 | 0.105 | 0.016 | 1.40E-10 | 43.1 | 0.46% |
| ER- BC | rs405343 | 16 | G | T | 0.38 | -0.118 | 0.022 | 4.70E-08 | 28.8 | 0.66% |
| ER- BC | rs28834106 | 19 | T | C | 0.29 | 0.129 | 0.02 | 3.80E-11 | 41.6 | 0.69% |
| ER- BC | rs9591325 | 13 | T | C | 0.96 | 0.212 | 0.034 | 4.20E-10 | 38.9 | 0.35% |
| ER- BC | rs6032662 | 20 | C | T | 0.02 | 0.134 | 0.018 | 2.80E-13 | 55.4 | 0.07% |
| ER- BC | rs6990534 | 8 | A | G | 0.7 | -0.107 | 0.018 | 3.60E-09 | 35.3 | 0.48% |
| ER- BC | rs35540610 | 2 | C | T | 0.26 | 0.135 | 0.019 | 2.90E-12 | 50.5 | 0.70% |
| ER- BC | rs9610458 | 22 | T | C | 0.35 | 0.114 | 0.017 | 4.60E-12 | 45.0 | 0.59% |
| ER- BC | rs1465697 | 19 | C | T | 0.31 | -0.124 | 0.019 | 3.50E-11 | 42.6 | 0.66% |
| ER- BC | rs72928038 | 6 | G | A | 0.02 | -0.161 | 0.025 | 9.00E-11 | 41.5 | 0.10% |
| ER- BC | rs11749040 | 5 | G | A | 0.23 | -0.197 | 0.023 | 3.50E-17 | 73.4 | 1.37% |
| ER- BC | rs17724508 | 16 | T | C | 0.96 | 0.213 | 0.038 | 2.60E-08 | 31.4 | 0.35% |
| ER- BC | rs9992763 | 4 | T | G | 0.84 | -0.09 | 0.016 | 4.50E-08 | 31.6 | 0.22% |
| ER- BC | rs35703946 | 16 | G | A | 0.89 | 0.173 | 0.029 | 1.90E-09 | 35.6 | 0.59% |
| ER- BC | rs2150879 | 17 | G | A | 0.9 | 0.104 | 0.016 | 3.30E-10 | 42.3 | 0.19% |
| ER- BC | rs59655222 | 1 | T | C | 0.85 | 0.123 | 0.019 | 3.80E-11 | 41.9 | 0.39% |
| ER- BC | rs28703878 | 8 | A | G | 0.57 | -0.134 | 0.021 | 4.50E-10 | 40.7 | 0.88% |
| ER- BC | rs1323292 | 1 | A | G | 0.98 | 0.124 | 0.022 | 1.40E-08 | 31.8 | 0.06% |
| ER- BC | rs13327021 | 3 | C | T | 0.28 | -0.113 | 0.017 | 4.50E-11 | 44.2 | 0.51% |
| ER- BC | rs12133753 | 1 | C | T | 0.83 | 0.128 | 0.022 | 8.50E-09 | 33.9 | 0.46% |
| ER- BC | rs12925972 | 16 | C | T | 0.48 | 0.095 | 0.017 | 3.10E-08 | 31.2 | 0.45% |
| ER- BC | rs35486093 | 1 | A | G | 0.15 | -0.179 | 0.028 | 1.60E-10 | 40.9 | 0.82% |
| ER- BC | rs2248137 | 20 | G | C | 0.26 | -0.112 | 0.017 | 7.80E-11 | 43.4 | 0.48% |
| ER- BC | rs1112718 | 10 | A | G | 0.55 | 0.106 | 0.017 | 2.50E-10 | 38.9 | 0.56% |
| ER- BC | rs1738074 | 6 | T | C | 0.32 | -0.114 | 0.017 | 9.90E-12 | 45.0 | 0.57% |
| ER- BC | rs7855251 | 9 | T | C | 0.38 | 0.11 | 0.02 | 4.20E-08 | 30.3 | 0.57% |
| ER- BC | rs12434551 | 14 | A | T | 0.66 | 0.104 | 0.016 | 1.80E-10 | 42.3 | 0.49% |
| ER- BC | rs57116599 | 2 | A | G | 0.82 | -0.12 | 0.02 | 2.60E-09 | 36.0 | 0.43% |
| ER- BC | rs10936602 | 3 | T | C | 0.76 | 0.108 | 0.019 | 1.70E-08 | 32.3 | 0.43% |
| ER- BC | rs7222450 | 17 | A | G | 0.43 | 0.098 | 0.017 | 1.80E-08 | 33.2 | 0.47% |
| ER- BC | rs802730 | 6 | T | C | 0.9 | 0.114 | 0.018 | 3.20E-10 | 40.1 | 0.23% |
| ER- BC | rs9843355 | 3 | G | A | 0.81 | 0.134 | 0.021 | 4.70E-10 | 40.7 | 0.55% |
| ER- BC | rs140522 | 22 | C | T | 0.53 | -0.111 | 0.018 | 2.80E-10 | 38.0 | 0.61% |
| ER- BC | rs9955954 | 18 | G | A | 0.74 | -0.11 | 0.019 | 1.50E-08 | 33.5 | 0.47% |
| ER- BC | rs6496663 | 15 | A | C | 0.67 | -0.101 | 0.018 | 2.80E-08 | 31.5 | 0.45% |
| ER- BC | rs1077667 | 19 | C | T | 0.88 | 0.152 | 0.021 | 8.40E-13 | 52.4 | 0.49% |
| ER- BC | rs4325907 | 3 | T | C | 0.46 | -0.099 | 0.017 | 3.70E-09 | 33.9 | 0.49% |
| ER- BC | rs55858457 | 7 | G | T | 0.45 | -0.113 | 0.02 | 1.20E-08 | 31.9 | 0.63% |
| ER- BC | rs11256593 | 10 | T | C | 0.13 | 0.186 | 0.017 | 6.80E-27 | 119.7 | 0.78% |
| ER- BC | rs12147246 | 14 | G | A | 0.84 | -0.099 | 0.017 | 4.30E-09 | 33.9 | 0.26% |
| ER- BC | rs483180 | 1 | G | C | 0.9 | -0.108 | 0.018 | 1.80E-09 | 36.0 | 0.21% |
| ER- BC | rs4808760 | 19 | G | C | 0.81 | -0.135 | 0.019 | 4.80E-13 | 50.5 | 0.56% |
| ER- BC | rs6670198 | 1 | C | T | 0.22 | -0.145 | 0.018 | 2.00E-16 | 64.9 | 0.72% |
| ER- BC | rs354033 | 7 | G | A | 0.78 | 0.108 | 0.019 | 1.20E-08 | 32.3 | 0.40% |
| ER- BC | rs12365699 | 11 | G | A | 0.98 | 0.144 | 0.023 | 3.10E-10 | 39.2 | 0.08% |
| ER- BC | rs438613 | 3 | C | T | 0.11 | 0.138 | 0.017 | 9.40E-17 | 65.9 | 0.37% |
| ER- BC | rs4896153 | 6 | A | T | 0.33 | -0.138 | 0.019 | 1.60E-13 | 52.8 | 0.84% |
| ER- BC | rs11079784 | 17 | C | T | 0.32 | 0.107 | 0.016 | 4.30E-11 | 44.7 | 0.50% |
| ER- BC | rs1250551 | 10 | G | T | 0.12 | -0.116 | 0.017 | 2.70E-11 | 46.6 | 0.28% |
| ER- BC | rs6589706 | 11 | A | G | 0.07 | 0.104 | 0.017 | 2.20E-09 | 37.4 | 0.14% |
| ER- BC | rs2317231 | 1 | G | T | 0.38 | 0.101 | 0.017 | 1.90E-09 | 35.3 | 0.48% |
| ER- BC | rs1177228 | 2 | G | A | 0.92 | 0.107 | 0.019 | 8.60E-09 | 31.7 | 0.17% |
| ER- BC | rs12478539 | 2 | G | C | 0.8 | 0.123 | 0.019 | 4.40E-11 | 41.9 | 0.48% |
| ER- BC | rs10063294 | 5 | A | G | 0.21 | -0.099 | 0.016 | 1.10E-09 | 38.3 | 0.33% |

ER: estrogen receptor. BC: Breast cancer. Chr: chromosome. EA: effect allele. EAF: effect allele frequency. OA: other allele. SE: standard error. SNP: single nucleotide polymorphism.
