# Supplementary material for: Multiple sclerosis and breast cancer risk: a meta-analysis of observational and Mendelian randomization studies
Source: Front Neuroinform. 2023 May 3;17:1154916. doi: 10.3389/fninf.2023.1154916 (PMC10191210; doi:10.3389/fninf.2023.1154916)
Supplement: Supplementary file 2 [file Table_2.DOCX]

**Supplementary Table 2.** Information on instrumental variables of MS from FinnGen database.

| Outcome | SNP | CHR | POS | EA | OA | BETA | EAF | SE | P | F | R^2^ |
| --- | --- | --- | --- | --- | --- | --- | --- | --- | --- | --- | --- |
| Overall BC | rs10947501 | 6 | 34413954 | G | A | 0.381 | 0.13 | 0.070 | 4.64E-08 | 29.6 | 3.28% |
| Overall BC | rs112723370 | 6 | 31433809 | G | T | 0.857 | 0.05 | 0.110 | 7.37E-15 | 60.7 | 6.98% |
| Overall BC | rs115272033 | 6 | 31850807 | C | T | 0.645 | 0.05 | 0.101 | 1.88E-10 | 40.8 | 3.95% |
| Overall BC | rs141298848 | 3 | 33988087 | T | C | 0.805 | 0.03 | 0.145 | 2.67E-08 | 30.8 | 3.77% |
| Overall BC | rs183697542 | 6 | 30846941 | T | C | 1.427 | 0.02 | 0.193 | 1.51E-13 | 54.7 | 7.98% |
| Overall BC | rs743771 | 6 | 32976909 | A | C | -0.305 | 0.46 | 0.047 | 9.87E-11 | 42.1 | 4.62% |
| Overall BC | rs9258981 | 6 | 29841195 | G | A | -0.337 | 0.53 | 0.048 | 2.64E-12 | 49.3 | 5.66% |
| Overall BC | rs9271069 | 6 | 32575700 | G | A | -1.143 | 0.86 | 0.071 | 1.54E-58 | 259.2 | 31.46% |
| Overall BC | rs9276832 | 6 | 32832400 | T | C | -0.305 | 0.29 | 0.053 | 6.38E-09 | 33.1 | 3.83% |
| Overall BC | rs9357119 | 6 | 31229747 | T | G | -0.316 | 0.39 | 0.046 | 8.31E-12 | 47.2 | 4.75% |
| ER+ BC | rs10947501 | 6 | 34413954 | G | A | 0.381 | 0.13 | 0.070 | 4.64E-08 | 29.6 | 3.28% |
| ER+ BC | rs112723370 | 6 | 31433809 | G | T | 0.857 | 0.05 | 0.110 | 7.37E-15 | 60.7 | 6.98% |
| ER+ BC | rs115272033 | 6 | 31850807 | C | T | 0.645 | 0.05 | 0.101 | 1.88E-10 | 40.8 | 3.95% |
| ER+ BC | rs141298848 | 3 | 33988087 | T | C | 0.805 | 0.03 | 0.145 | 2.67E-08 | 30.8 | 3.77% |
| ER+ BC | rs183697542 | 6 | 30846941 | T | C | 1.427 | 0.02 | 0.193 | 1.51E-13 | 54.7 | 7.98% |
| ER+ BC | rs743771 | 6 | 32976909 | A | C | -0.305 | 0.46 | 0.047 | 9.87E-11 | 42.1 | 4.62% |
| ER+ BC | rs9258981 | 6 | 29841195 | G | A | -0.337 | 0.53 | 0.048 | 2.64E-12 | 49.3 | 5.66% |
| ER+ BC | rs9271069 | 6 | 32575700 | G | A | -1.143 | 0.86 | 0.071 | 1.54E-58 | 259.2 | 31.46% |
| ER+ BC | rs9276832 | 6 | 32832400 | T | C | -0.305 | 0.29 | 0.053 | 6.38E-09 | 33.1 | 3.83% |
| ER+ BC | rs9357119 | 6 | 31229747 | T | G | -0.316 | 0.39 | 0.046 | 8.31E-12 | 47.2 | 4.75% |
| ER- BC | rs10947501 | 6 | 34413954 | G | A | 0.381 | 0.13 | 0.070 | 4.64E-08 | 29.6 | 3.28% |
| ER- BC | rs112723370 | 6 | 31433809 | G | T | 0.857 | 0.05 | 0.110 | 7.37E-15 | 60.7 | 6.98% |
| ER- BC | rs115272033 | 6 | 31850807 | C | T | 0.645 | 0.05 | 0.101 | 1.88E-10 | 40.8 | 3.95% |
| ER- BC | rs141298848 | 3 | 33988087 | T | C | 0.805 | 0.03 | 0.145 | 2.67E-08 | 30.8 | 3.77% |
| ER- BC | rs183697542 | 6 | 30846941 | T | C | 1.427 | 0.02 | 0.193 | 1.51E-13 | 54.7 | 7.98% |
| ER- BC | rs743771 | 6 | 32976909 | A | C | -0.305 | 0.46 | 0.047 | 9.87E-11 | 42.1 | 4.62% |
| ER- BC | rs9258981 | 6 | 29841195 | G | A | -0.337 | 0.53 | 0.048 | 2.64E-12 | 49.3 | 5.66% |
| ER- BC | rs9271069 | 6 | 32575700 | G | A | -1.143 | 0.86 | 0.071 | 1.54E-58 | 259.2 | 31.46% |
| ER- BC | rs9276832 | 6 | 32832400 | T | C | -0.305 | 0.29 | 0.053 | 6.38E-09 | 33.1 | 3.83% |
| ER- BC | rs9357119 | 6 | 31229747 | T | G | -0.316 | 0.39 | 0.046 | 8.31E-12 | 47.2 | 4.75% |

ER: estrogen receptor. BC: Breast cancer. Chr: chromosome. EA: effect allele. EAF: effect allele frequency. OA: other allele. SE: standard error. SNP: single nucleotide polymorphism.
