# Supplementary figures and images for: Multiple sclerosis and breast cancer risk: a meta-analysis of observational and Mendelian randomization studies
Source: Front Neuroinform. 2023 May 3;17:1154916. doi: 10.3389/fninf.2023.1154916 (PMC10191210; doi:10.3389/fninf.2023.1154916)

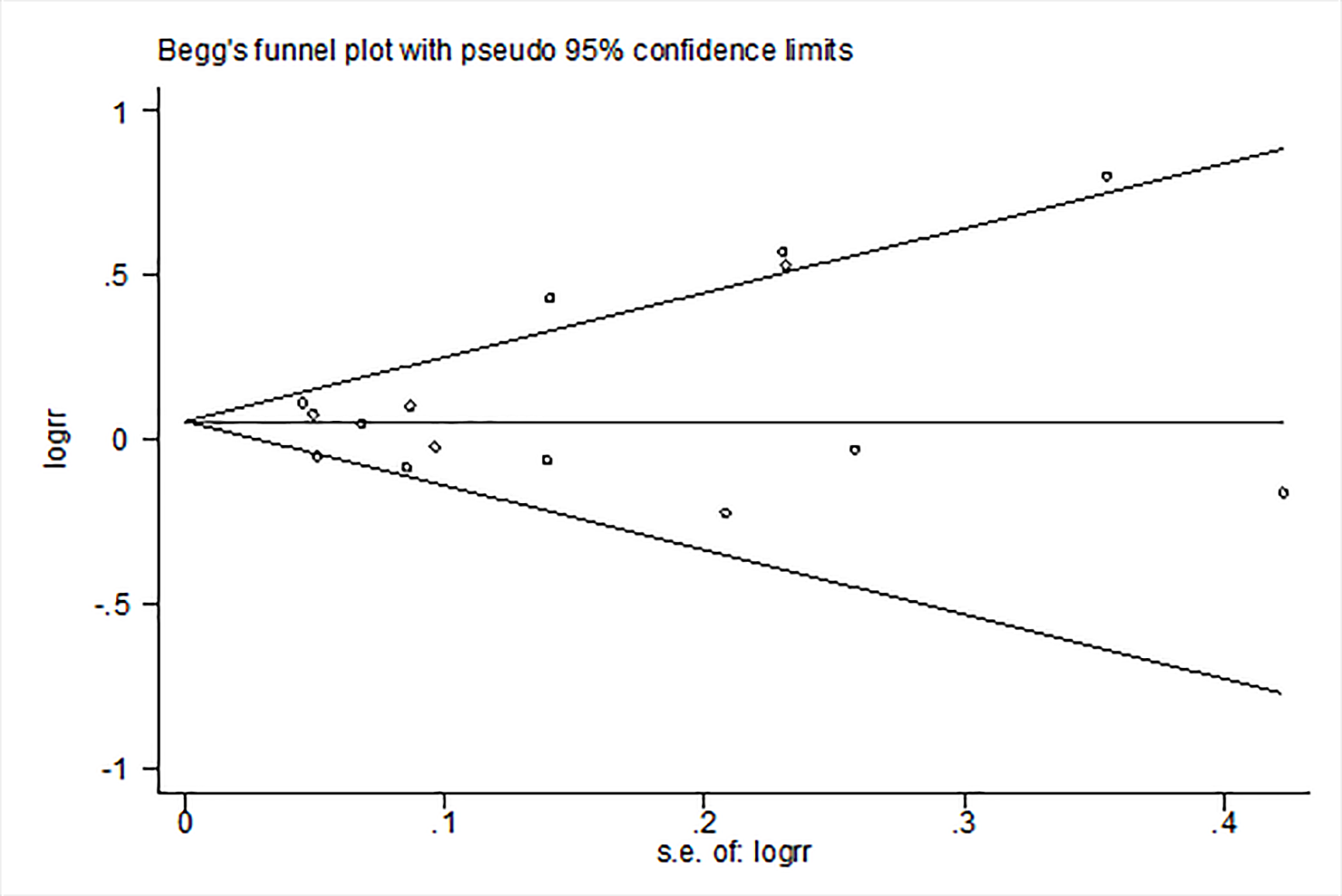

Supplement: Supplementary Figure 1 — Funnel plot of Begg’s test for publication bias. [file Image_1.TIF]

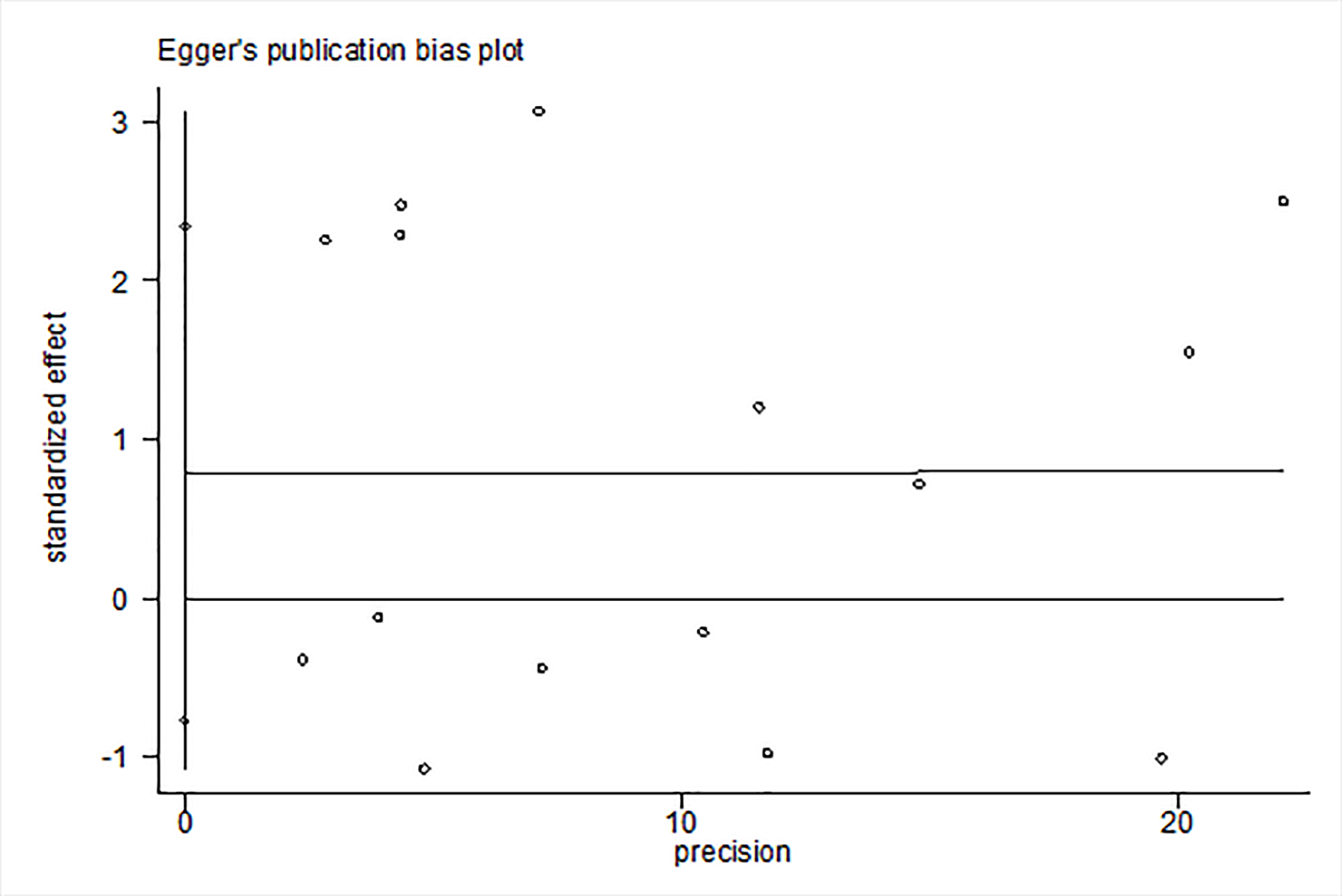

Supplement: Supplementary Figure 2 — Funnel plot of Egger’s test for publication bias. [file Image_2.TIF]

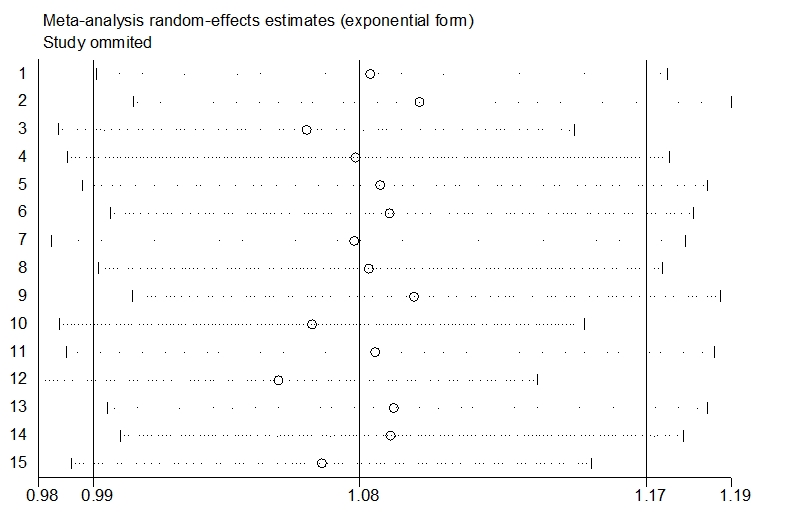

Supplement: Supplementary Figure 3 — Sensitivity analysis of the leave-one-out test for the meta-analysis. [file Image_3.JPEG]
